# Engaging Health Systems to Increase Colorectal Cancer Screening: Community–Clinical Outreach in Underserved Areas of Wisconsin

**DOI:** 10.5888/pcd10.130180

**Published:** 2013-11-21

**Authors:** Noelle K. LoConte, Lauren Weeth-Feinstein, Amy Conlon, Sheryl Scott

**Affiliations:** Author Affiliations: Lauren Weeth-Feinstein, Amy Conlon, University of Wisconsin Carbone Cancer Center and Wisconsin Comprehensive Cancer Control Program, Madison, Wisconsin; Sheryl Scott, Scott Consulting Partners, Richland Center, Wisconsin.

## Abstract

**Background:**

Colorectal cancer is the fourth most commonly diagnosed cancer and the second leading cause of cancer-related death in Wisconsin. Incidence and mortality rates for colorectal cancer vary by age, race/ethnicity, geography, and socioeconomic status. From 2010 through 2012, the Wisconsin Comprehensive Cancer Control Program awarded grants to 5 regional health systems for the purpose of planning and implementing events to increase colorectal cancer screening rates in underserved communities.

**Community Context:**

Grantees were chosen for their ability to engage community partners in reaching underserved groups including African American, Hispanic/Latino, Hmong, rural, and uninsured populations in their service areas.

**Methods:**

Grantees identified target populations for proposed screening events, designated institutional planning teams, engaged appropriate local partner organizations, and created plans for follow-up. All grantees implemented 1 or more colorectal cancer screening events within 6 months of receiving their awards. Events were conducted in 2 phases.

**Outcomes:**

Participating health systems organized 36 screening events and distributed 633 individual test kits; 506 kits were returned, of which 57 (9%) tested positive for colorectal abnormalities. Of attendees who received screening, 63% were uninsured or underinsured, 55% had no previous screening, 46% were of a racial/ethnic minority group, 22% had a family history of cancer, and 13% were rural residents. This project strengthened partnerships between health systems and local organizations.

**Interpretation:**

An effective strategy for improving colorectal cancer screening rates, particularly among underserved populations, is to award health systems grants for implementing community-based screening events in conjunction with community partners.

## Background

Colorectal cancer (CRC) is the fourth most commonly diagnosed cancer in Wisconsin and the second leading cause of cancer-related death (1). Statewide incidence and mortality rates for CRC vary by race and ethnicity; African Americans, American Indians, and certain Asian populations are more likely than whites to develop this disease and die from it (2). CRC disparities are related to the effect geography and socioeconomic status have on obtaining proper cancer screenings. Rural, low-income, and uninsured people are less likely to receive and be up-to-date with CRC screening (3,4).

CRC screening guidelines recommend a regular fecal occult blood test, sigmoidoscopy, or colonoscopy from age 50 until age 75; however, less than two-thirds of Americans aged 50 or older follow those recommendations (5,6).

More than 30% of eligible Wisconsin adults have never had a colonoscopy or sigmoidoscopy (7). Survival rates for CRC can exceed 90% if diagnosed early, yet over half of recent cases in Wisconsin were diagnosed at a regional or distant stage, indicating a need for earlier detection through improved screening programs (8).

The Wisconsin Comprehensive Cancer Control Program (WCCCP) works to engage public, private, and community partners to implement a statewide approach to cancer control (9). To examine possibilities for improving CRC screening statewide, WCCCP convened a stakeholder group, the Wisconsin Colorectal Cancer Screening Taskforce, which analyzed county-level screening rates and assessed current screening capacity. Those results guided the subsequent development of a grant application to fund health systems’ development of CRC screening events for underserved Wisconsin communities.

From 2010 through 2012, WCCCP awarded grants to 5 regional health systems for the purpose of planning and implementing CRC screening events. Health systems and community partners designated underserved populations in their service areas and then designed events to maximize participation for those attendees.

## Community Context

Wisconsin is a geographically varied state that includes urban, rural, and tribal areas. Although most of the state’s population is non-Hispanic white, there are regional concentrations of racial and ethnic minorities: 6.5% African American, 6.1% Hispanic or Latino, 2.4% Asian (33% of whom are Hmong), and 1.1% American Indian (10).

Disparities in CRC incidence and mortality between African Americans and whites in Wisconsin are large and worsened between 1995 and 2006 (11). Low socioeconomic status is associated with higher incidence of and poorer survival from CRC, and 12% of Wisconsin’s population lives below the poverty line (10,12). Nearly one-third of Wisconsinites also live in rural areas, where cancer screening and early detection services are frequently underused (13,14). In 2009, WCCCP conducted an endoscopic capacity assessment in Wisconsin and found that, although capacity to provide CRC screening to all residents of Wisconsin existed, distances, cost, and time from work were barriers to receiving cancer screenings for rural residents (14).

The objective of WCCCP’s CRC screening grants program was to reduce disparities by increasing CRC screening rates in underserved populations in Wisconsin. Our community engagement objective was to strengthen partnerships between regional health care systems, community partners, and target populations in their communities. We hoped that as an outcome of our community engagement efforts, grantees’ experiences would serve as examples for other health systems interested in increasing CRC screening rates in underserved populations.

## Methods

Our community-based grant program enabled Wisconsin health systems and their community partners to develop and implement new CRC screening events for underserved populations in their respective service areas. We chose to use this partner approach to engage target populations because health systems have the technical capacity to offer screening and follow-up in a community setting, and local community partners understand the targeted populations and can offer grassroots outreach. Community–clinical partnerships are an essential element of this program.

WCCCP awarded grants to 5 health care systems with access to target populations whose demographic, geographic, or economic characteristics limit their access to CRC screening services. System A and System B are both urban-based health systems that serve a large number of African American and uninsured patients. System C spans a small city and surrounding rural geographic area containing a large immigrant Hmong community, and System D is a suburban health system that primarily serves a single county with many rural and Hispanic residents. System E is a rural, federally qualified health center (FQHC) (**Table 1**).

**Table 1:** Summary of Wisconsin Health Systems Given Grants for Colorectal Cancer Screening Events, Targeted Local Populations, and Types of Community Partners Assisting With Event Planning and Implementation, 2010–2012

Grantee	System A	System B	System C	System D	System E
Health system characteristic	Urban, integrated	Urban, integrated	Suburban/rural, integrated	Suburban/rural, community-based	Rural, FQHC
Local target population(s) for colorectal cancer screening	Low-income; urban; African American; Hispanic/Latina	Low-income; urban and rural	Hmong elders; veterans; retirees/seniors; low-income; rural	Hispanic/Latina; low-income; rural	Low-income; rural
Community partner organization	Local Well Woman Program offices	Local American Cancer Society office; local Well Woman Program offices	Hmong community groups; local governmental agencies; local Well Woman Program office	Various Hispanic community organizations; local Well Woman Program office; local American Cancer Society office; county extension office	Area school district; free local health clinic; county extension office

Abbreviation: FQHC, federally qualified health center.

Applications for this funding opportunity were solicited via several channels: an e-mail listserv, monthly newsletters, and direct recruitment by program staff. Applicants were asked to clearly identify a target population for their proposed event or events and explain the need for increased CRC screening. Preference was given to proposals that included access to free screenings for uninsured or underinsured populations. Applicants designated an institutional planning team, led by a screening event coordinator. Grant applications also included a budget, a timeline for implementation, and an evaluation plan.

Another important aspect of the application process was identification of community partners: local organizations or leaders outside of each health system who would be able to help with participant recruitment and event design and implementation. Health systems selected their own community partner or partners. The selection was made on the basis of past partnerships with the community partner. WCCCP reviewed applications for community partners’ commitment to the proposed event and successful previous engagement in cancer screening efforts. Finally, a CRC screening event requires staff to organize the event, a facility where the screening will occur, screening kits, access to patients, CRC educational materials, and capacity for follow-up with participants. Our grantees and their community partners were able to secure all of these additional resources.

Within 6 months of receiving funding, each award recipient was expected to develop and implement 1 or more CRC screening events. Grantees could create free-standing events or integrate screening activities into an existing event. Because of Centers for Disease Control and Prevention (CDC) regulations, grant money could not be used for direct patient services; all participating health care systems were therefore required to provide CRC screening test kits and documentation of available funds for additional diagnosis in case of a positive screening result in an uninsured or underinsured patient. All the participating health systems chose to offer immunochemical fecal occult blood tests (iFOBTs) as their screening method at their events. On the basis of published data, a 3% positive FOBT rate was anticipated (15).

The CRC screening events were conducted in 2 phases. Phase I began in October 2010. Throughout, grantees shared their progress by submitting monthly planning meeting minutes. Grantees were also required to participate in monthly teleconference calls to discuss progress and challenges. Events were advertised by using various promotional media developed in conjunction with community partners, including clinic-based flyers, mailed postcards, and Spanish-language radio ads. Most Phase I events took place in March 2011.

All grantees participated in process and outcome evaluations of their event or events. Evaluation methods used included event observations, interviews with key health system staff and their community partners, and screening forms that participants completed when they received their screening kit. Health systems also participated in monthly calls to discuss the planning process and completed final reports for both Phases I and II. WCCCP provided each of the grantees with a summary of Phase I evaluation results, including a “menu of ideas” for how they might improve future events. Phase I grantees were encouraged to tailor their original strategies on the basis of lessons learned and to apply for another round of funding.

Four of the 5 original grantees submitted applications and received funding in Phase II, which began in October 2011. The main purpose of Phase II was to refine previous events and test new ideas to bring community partners and health systems together to reach underserved populations with CRC screening. Most events in Phase II were held in February and March of 2012.

Our final project evaluation was based on multiple objectives: maximum community participation at events through increased CRC screening and the creation of community–clinical partnerships. WCCCP employed both process and outcome evaluation methods and collaborated with an external evaluator to review and synthesize evaluation data into 2 final reports. At the conclusion of each grant phase, the evaluator used qualitative and quantitative data to summarize the event planning process.

We collected information from individual event sites to learn as much about the implementation process as possible. Our evaluator created a database for grantees to track patients who received test kits at screening events. To track community representation, registration sheets at each event asked for demographic characteristics of the person being offered CRC screening. Additionally, either the external evaluator or WCCCP staff attended at least 1 of each grantee’s events and conducted an onsite observational assessment. We created a brief Internet survey for community partners’ representatives to solicit partnership ratings and comments for improvement, which received 27 responses — each of the 5 health systems recruited 1 or more community partners, and sometimes multiple people represented the community partners (16 from Phase I and 11 from Phase II). We also conducted telephone interviews with 2 additional partners who preferred that format for feedback. All but 2 community partners’ representatives stated they would definitely collaborate with a health system on future events. Several representatives provided ideas for other health-related events they would like to see in their areas.

## Outcomes

The health systems in our grant program offered 36 CRC screening events in Phase I and II from 2010 through 2012; System A, which only participated in Phase I, did an evening presentation to participants in the Wisconsin Well Woman Program (the state’s National Breast and Cervical Cancer Early Detection Program), and Systems B and C also did events with the Wisconsin Well Woman Program participants in Phase II. System B also staffed 14 outreach booths at their urban clinics in Phase I. In Phase I, System C did 6 community health events, including 1 booth at an annual community health fair for Hmong elders. In Phase II, health systems focused on just 2 events: a booth at the Hmong Elder health fair and the Wisconsin Well Woman Program event. System D did a Hispanic cancer prevention fair held at a hospital in both Phases I and II. System E held 2 events for school district staff and an outreach booth at a health mission for Phase I, and in Phase II they had outreach booths at 5 previously scheduled country agricultural extension events (**Table 2** **).** CRC screening kits were distributed at all of the events. All of these events were new or the screening component of the event was new, and the 5 health systems did not distribute iFOBT kits to these populations before this effort. Overall, systems differed in their approaches to planning and implementation, and resulting events varied widely in terms of number of screening kits distributed, percentage returned, and percentage testing positive for abnormalities.

**Table 2:** Summary of All Colorectal Cancer Screening Events Developed by Participating Wisconsin Health Systems, With Screening Test Kit Distribution Totals, 2010–2012

Grantee	System A	System B	System C	System D	System E
Type of screening event	Special event for invited participants	Outreach booths at multiple clinics; special events for invited participants	Outreach booth at Hmong health event; special event for invited participants	Comprehensive Hispanic health fair	Outreach booths at clinic and existing community events
Total no. of events	1 (phase I only)	17	8	2	8
Total no. of test kits distributed	105	143	77	257	51
Total no. of test kits returned	101	98	52	216	39
Incentives given to those who return kits	None	$10 grocery gift card	$5 gas gift card	$15 cash	$10 cash
Total no. of positive tests	7	6	5	33	6

With the primary goals of the grant project in mind, 3 key outcomes were assessed: event participation by members of underserved communities, distribution and return of CRC screening test kits, and effectiveness of community–clinical partnerships.

### Event participation by underserved communities

**Figure 1:**
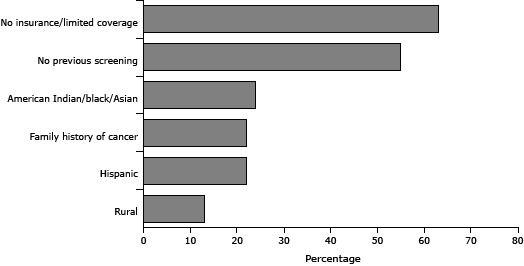
Sociodemographic characteristics of eligible Wisconsin adults screened for colorectal cancer in conjunction with the Wisconsin Comprehensive Cancer Control Program screening events grant project, as a percentage of the total number offered screening. Participants (n = 633) may be counted in more than 1 category. **Table:** 

Our program resulted in the distribution of more than 600 iFOBT kits to underserved Wisconsin residents. Of all event attendees who received test kits (n = 633), 63% reported that they were uninsured or underinsured, 55% had no previous screening, 46% belonged to a racial/ethnic minority group, 22% reported a family history of cancer, and 13% were rural residents (**Figure 1** **).** Most of those reached had not been offered screening otherwise.

Characteristic	% of Total Screened
No insurance/limited coverage	63
No previous screening	55
American Indian/black/Asian	24
Family history of cancer	22
Hispanic	22
Rural	13

### Distribution and return of CRC screening test kits

**Figure 2:**
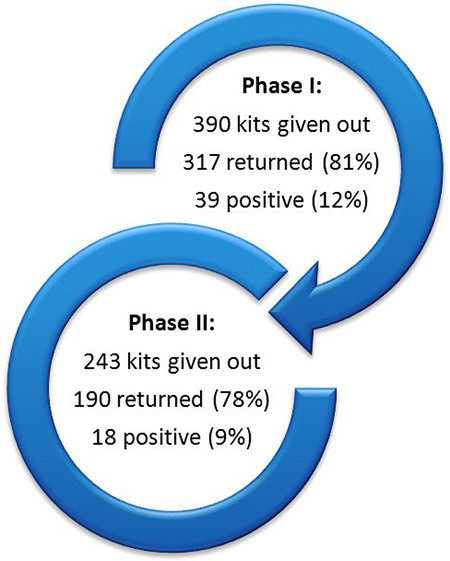
Total fecal immunochemical blood test (iFOBT) kits distributed, returned, and testing positive for abnormalities in Phases I and II of the Wisconsin Comprehensive Cancer Control Program colorectal cancer screening events grant project.

Our program contributed to an increase in CRC screening in the underserved populations in the catchment areas of the participating health systems. The 36 screening events we funded enabled the distribution of 633 CRC screening kits to a range of underserved community members throughout Wisconsin. Of those kits, 506 (80%) were returned to local health systems. These return rates are higher than what has been reported previously in the literature (25%–63%) (16–18). Sites that used both incentives and follow-up calls had the highest return rates. Fifty-seven (9%) of the returned iFOBTs tested positive for colorectal abnormalities, which was higher than the initial estimate of 3% (15) (**Figure 2**). Of the 57 who tested positive, all but 5 were able to be reached and given further diagnostic testing and care as needed. The database created by our external evaluator, which included contact information for the iFOBT kit recipients, made it easier for health systems to track the distribution and return of screening test kits.

### Effectiveness of community–clinical partnerships

Our program facilitated new and productive linkages between 5 major health systems and various community partners, including Hispanic and Hmong community resource networks, county agricultural extension offices, the American Cancer Society, the Wisconsin Well Woman Program, aging and disability centers, a veteran services office, a local school district, and low-income clinics. The high level of community partner participation in planning and implementing the various screening events (as measured in the community partner evaluation survey) is a reflection of grantees’ meaningful collaboration with relevant community partners. Furthermore, grantees’ and their community partners’ ongoing participation in our program evaluation process invited mutual reflection about how to strengthen community–clinical partnerships, resulting in a more concretely focused set of events in Phase II.

It was important for health systems and community partners to take time to learn how the other conducts its work and for both to assume responsibility for the event. Survey results from community partners indicated that it was helpful for health systems to find a balance between asking too much of the community partner and not collaborating enough or underusing community expertise for outreach. Events with more balanced clinical–community partnerships yielded higher participation, higher rates of return on screening tests, and better survey ratings from community partners.

### Factors that facilitate efficient and effective CRC screening events

The use of qualitative process evaluation, such as event observations, helped identify several factors that may influence the success of cancer screening events. For example, we observed that stand-alone screening events planned by health systems and community partners were the most effective type of venue for distributing test kits and ensuring their return. Grantees who changed to this type of event in Phase II felt that they were more efficient in reaching the target populations when they compared their approach with that in Phase I. One site gave out the same number of kits (n = 71) in both phases of the project, but in Phase I, they did so with booths at 14 events versus holding a one-time stand-alone event in Phase II. We also learned that return rates for test kits were generally highest when supported by follow-up calls and incentives, such as a grocery or gas gift card or cash.

### Challenges

Although this project generated many positive outcomes, there were some challenges. Follow-up on positive screening tests was not always easy. In Phase I, the Hmong community members we initially screened had a disproportionately high number of positive results, yet we were unable to reach most of those people for follow-up. Difficulty in reaching Hmong members with positive test results persisted in Phase II, when additional resources for interpreters were included. Additional research is needed to understand the reasons for higher positive rates among this population and to determine the most effective strategies for follow-up. Another issue in Phase I was that some systems struggled to secure resources for follow-up diagnostic testing (in cases of uninsured or underinsured patients with an initial positive iFOBT screening); we clarified this expectation in our Phase II application process, and applicants who were not able to provide advance guarantees of adequate support were excluded from participating in Phase II. We also observed that more resources may be required to reach rural participants since they are fewer in number and farther apart geographically.

## Interpretation

We believe this grant program was worthwhile, because of its ability to galvanize community–clinical partnerships and reach underserved populations with much-needed cancer screening. Lessons learned from this program are relevant to health systems wanting to offer cancer screenings to underserved populations, particularly with regard to considerations such as event settings, community partnerships, participant recruitment, and follow-up.

Holding a stand-alone event enabled distribution of higher numbers of tests than adding events to a pre-existing forum. However, in rural areas, special events still need to be tested as venues for screening to ensure that the target audience will be in attendance and be receptive to getting screened.

The choice of setting also affects recruitment; for example, grantees reported that it took more time and planning than anticipated to get staff and patients involved in screenings at busy low-income clinics. In all settings, recruitment may be boosted by the use of promotional mailings. Sites that sent out personalized letters in advance of events had a strong, positive response from community members targeted for screening.

To improve the success of community–clinical partnerships, clear roles and responsibilities among the partners should be delineated from the start. Community partners bring their relationships and expertise to tailor programming to the needs of local populations, and health systems can contribute clinical resources and knowledge to support the implementation of quality care services.

Budgeting adequate staff time to follow up on positive screening tests is another important element of completing a successful screening project. In particular, members of underserved minority groups may require dedicated and culturally competent outreach to ensure that they receive notification of their test results and adequate diagnostic follow-up.

Awarding grants to health systems for the purpose of implementing community screening events was an effective strategy for improving rates of CRC screening in Wisconsin, particularly in underserved populations. This program strengthened partnerships between health care systems and local organizations, both of which have ongoing potential to benefit the partners and participants involved.
